# Estimated prevalence of Niemann–Pick type C disease in Quebec

**DOI:** 10.1038/s41598-021-01966-0

**Published:** 2021-11-19

**Authors:** Marjorie Labrecque, Lahoud Touma, Claude Bhérer, Antoine Duquette, Martine Tétreault

**Affiliations:** 1 Bioinformatics Program, Department of Biochemistry and Molecular Medicine, Université de Montréal, Montréal, QC Canada; 2 CHUM Research Center, Tour Viger, 900 rue Saint-Denis, R, Montréal, QC H2X 0A9 Canada; 3 Department of Neurosciences, Université de Montréal, Montréal, QC Canada; 4 Department of Human Genetics, McGill University, Montréal, Canada; 5 Neurology Service, Department of Medecine, André-Barbeau Movement Disorders Unit, CHUM, Montréal, Canada; 6 Genetic Medicine Service, Department of Medecine, CHUM, 1000 rue Saint-Denis, Montréal, QC H2X 0C1 Canada

**Keywords:** Genetics, Neuroscience

## Abstract

Niemann–Pick type C (NP-C) disease is an autosomal recessive disease caused by variants in the *NPC1* or *NPC2* genes. It has a large range of symptoms depending on age of onset, thus making it difficult to diagnose. In adults, symptoms appear mainly in the form of psychiatric problems. The prevalence varies from 0.35 to 2.2 per 100,000 births depending on the country. The aim of this study is to calculate the estimated prevalence of NP-C in Quebec to determine if it is underdiagnosed in this population. The CARTaGENE database is a unique database that regroups individuals between 40 and 69 years old from metropolitan regions of Quebec. RNA-sequencing data was available for 911 individuals and exome sequencing for 198 individuals. We used a bioinformatic pipeline on those individuals to extract the variants in the *NPC1/2* genes. The prevalence in Quebec was estimated assuming Hardy–Weinberg Equilibrium. Two pathogenic variants were used. The variant p.Pro543Leu was found in three heterozygous individuals that share a common haplotype, which suggests a founder French-Canadian pathogenic variant. The variant p.Ile1061Thr was found in two heterozygous individuals. Both variants have previously been reported and are usually associated with infantile onset. The estimated prevalence calculated using those two variants is 0.61:100,000 births. This study represents the first estimate of NP-C in Quebec. The estimated prevalence for NP-C is likely underestimated due to misdiagnosis or missed cases. It is therefore important to diagnose all NP-C patients to initiate early treatment.

## Introduction

Lysosomal storage disorders, like Gaucher’s disease, Tay-Sachs disease or Niemann–Pick type C (NP-C) disease are a group of diseases characterized by cholesterol trafficking problems^1^. The collective prevalence of lysosomal storage disorders is 1:5.000 births^2^. NP-C (MIM 257220 and MIM 607625) are neurodegenerative autosomal recessive and pan-ethnic diseases with a prevalence varying between 0.35 and 2.2 per 100,000 births^3,4^. NP-C has different symptoms depending on the age of onset, which can be infantile, juvenile or in adolescence and adult-onset. The classical phenotype for NP-C is found in the infantile and juvenile populations^1^. The clinical presentation is neuro-visceral with hepatosplenomegaly and neurological signs like delay in motor skills, clumsiness, hypotonia and ataxia^5^. However, adult-onset subtypes have been reported and often present an atypical phenotype with psychiatric symptoms. In some cases, it can mimic other neurodegenerative diseases such as Alzheimer, Wilson or Parkinson^6–8^. The clinical heterogeneity observed in NP-C makes the diagnosis challenging and it is often delayed, especially in atypical subtypes. As a result, the number of misdiagnoses could be high, and this may affect the estimated prevalence.

NP-C is caused by pathogenic variants in the *NPC1* gene (NM_000271) in 95% of cases and the rest is due to pathogenic variants in the *NPC2* gene (NM_006432)^5^. In the gnomAD database, there are 2,227 variants in the *NPC1* gene and 467 in the *NPC2* gene (accessed on 7 April 2021) including intronic and UTR variants. An o/e (LOEUF) score above 0.35 for missense variants in both *NPC1* and *NPC2* suggests that these genes are somewhat tolerant to change and may be unlikely to have heterozygote pathogenic variants for the severe pediatric typical NP-C^9^. However, these values need to be interpreted with caution in the context of atypical or adult-onset NPC. The NPC-db2 database (see “Patients and methods” section) is also often used to assess the pathogenicity of variants. It regroups 692 variants in the *NPC1* gene and their classification. Amongst all those variants, 200 in *NPC1* and 5 in *NPC2* have been reported as pathogenic^10^. Furthermore, there is growing evidence that heterozygote carriers of recessive mutations can increase the risk of developing a disease and even lead to milder and adult-onset phenotypes^11,12^ including for neurodegenerative disorders such as Parkinson and NP-C^13^. Regarding NP-C specifically, one study described hepatosplenomegaly in 71% of heterozygotes carriers and several individuals had impaired cognitive functions^14^. The same study also suggests that NP-C heterozygosity may lead to late-onset neurodegeneration. Thus, the late-onset form of NP-C, due to heterozygous mutations or simply the high heterogeneity of symptoms, can be misdiagnosed and result in NP-C being underdiagnosed.

The prevalence of the disease is variable worldwide due to population differences, diagnostic awareness and diagnostic methodology^5,15^. Some populations may also have higher disease prevalence because of a founder effect, leading some rare variants to become more frequent. French-Canadians from Quebec are a well-known example of such founder population, with more than 30 monogenic conditions showing an increased prevalence and/or particular variants/phenotypes^16,17^. Among the French-Canadian population, we also observe regional founder effects such as in the Saguenay and Gaspesia regions^18^. A founder effect was previously reported in Canada for Niemann–Pick disease. Originally, Niemann–Pick type D (NP-D), had been reported clinically in Nova Scotia in four individuals of Acadian ancestry^19^. The founder pathogenic variant, p.Gly992Trp in *NPC1*, has a carrier frequency between 10 and 26%^20^. The high frequency of this variant is the result of a homogeneous genetic pool in the community and is unknown outside of Nova Scotia^19^. Since the identification of the underlying genetic abnormality, NP-D is no longer recognized as a different disease and it is now included in the NP-C spectrum^5^. Considering the founder effect in the French-Canadian population, we want to explore how it may affect the prevalence of rare variants in *NPC1* and *NPC2* and, consequently, of the NP-C disease. In Quebec, CARTaGENE (CaG) is a unique prospective cohort of healthy individuals^21^. It was developed to help study diseases and the genetics of the Quebec population.

Therefore, our goal is to measure the prevalence of NP-C in the Quebec population using population genetics data from CaG. Our results will inform on genetic testing approaches to be applied in a clinical setting in cases presenting clinical features reminiscent of NP-C and, more importantly, when an atypical form is encountered.

## Patients and methods

CaG contains genomic data and health information for 20,000 residents of Quebec between the age of 40 and 69^21^. The data used in our analysis comes from the RNA-sequencing (RNA-seq) of 911 people and from the exome-sequencing (exome-seq) of 198 people in the CaG cohort^21^. Among the 1,109 data sets, 93 people are in both the RNA-seq and exome-seq groups, which gives us sequencing information on 1,016 distinct individuals. The cohort used in this study is representative of Quebec’s population based on sex, age and ethnicity^22^. Individuals in our cohort had no known neurological diseases. The population structure was previously verified with a principal component analysis to confirm the French Canadians ancestry of the individuals of CaG^23^.

The use of RNA-sequencing to identify variants has been proven to be reliable and enable the identification of high-quality variants^24–27^.The pipeline for identification and classification of NP-C variants was used as described before^22^. Briefly, the sequences from the 1016 individuals were aligned to the human reference genome version GRCh37. The RNA-seq was aligned with HISAT2^28^ and the variants were identified with VarDict^29^. The exome-seq data was aligned with BWA^30^ and the variants were identified using GATK^31^. Both datasets were annotated with ANNOVAR^32^ as well as custom scripts. Annotations include different pathogenicity scores (CADD^33^, SIFT^34^, Polyphen2^35^), conservation scores (phastCons^36^ and GERP^37^) and allele frequencies of different databases (GnomAD^9^, NPC-db2 (https://medgen.medizin.uni-tuebingen.de/NPC-db2/ accessed on 18 July 2019)). We then kept the rare missense and indel variants with the following filters: allelic frequency (AF) < 1%, a pathogenicity score CADD > 15 and inversed SIFT or Polyphen2 > 0.75 and for conservation a GERP > 5 or phastCons > 500. The pathogenicity of variants identified in *NPC1* and *NPC2* was assessed using the ACMG guidelines^38^. Using this approach, two pathogenic variants were selected for further analysis. No evidence of allele degradation through nonsense mediated decay was observed in our cohort.

For each variant, we estimated the allele frequency in our cohort as the count of alternate allele divided by 2,032, the total number of alleles. The allele frequencies observed in our cohort were compared to that observed in exome and genome sequencing data from 55,852 Non-Finnish Europeans from the gnomAD v2.1 database^9^. To estimate which variants are found at significantly higher frequencies in the CaG cohort compared to Non-Finnish Europeans, we applied Fisher’s Exact test one-sided alternative as implemented in R version 4.1.0 to obtain the p-value and the odds ratio.

Summing allele frequencies over all pathogenic variants we obtained the cumulative frequency of pathogenic variants in *NPC1*/*2* genes. The prevalence was estimated using Hardy–Weinberg equation p^2^ + 2pq + q^2^ = 1, where q is the cumulative frequency of pathogenic variants. We can estimate q^2^, the frequency of homozygotes, as the expected birth incidence assuming that it is at Hardy–Weinberg equilibrium and that the penetrance is complete. We were then able to calculate the number of births that could be affected by NP-C every year in Quebec by multiplying q^2^ by the number of births in Quebec in 2019. By dividing the number of births in Quebec by the number of births that could be affected by NP-C we can measure the prevalence and report it per 100,000 births.

### Ethics approval

The Sample and Data Access Committee (SDAC) of CaG approved the use of the genetic and baseline characteristics for our study. All genetic and bioinformatic analysis were carried out in accordance with relevant guidelines and regulations. Our protocol was approved by our institution’s research ethics board (CR-CHUM REB, Project 18.116).

### Consent to participate

Informed consent was obtained from all individual participants included in the study.

## Results

### Identification of two *NPC1*/*2* pathogenic variants in the CaG cohort

In the CaG cohort, we found two variants classified as pathogenic in the *NPC1* gene (Table 1). The first one is in exon 10, c.1628C > T:p.Pro543Leu (P543L). This variant was found once before in a homozygote individual of French Canadian origin^39^. In our cohort, all three individuals with the P543L variant were found in the RNA-seq data and were heterozygote carriers of the variant. The first person identified with the P543L variant is a 46-year-old Caucasian woman. She has migraine occurrences but no known neurodegenerative disease. The second individual is a 51-year-old Caucasian male, also without known neurodegenerative disease. The third individual carrying the P543L variant, is a 62-year-old Caucasian woman also without neurodegenerative disease. All three individuals were sampled in the region of Quebec City, a region previously associated with founder mutations or diseases^16,40^. Thus, considering that all carriers of the P543L variant came from the same region in Quebec, we wanted to explore if they shared a haplotype. We found a haplotype of 9.4 Mb surrounding the variant that was shared among all carriers, suggesting a recent common ancestor, putatively of French-Canadian origin (Table 2).

**Table 1:** NP-C variants classified as pathogenic.

cDNA	Protein	Exon	Variant type	rsID	Allele number detected
c.1628C > T	p.P543L	10	missense	rs369368181	3
c.3182T > C	p.I1061T	21	missense	rs80358259	2

**Table 2:**
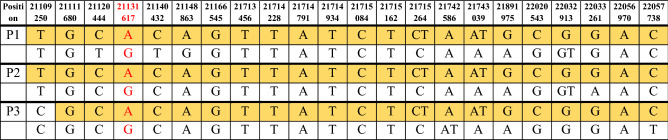
Haplotypes of individuals with the P543L variant (in red).

The second pathogenic variant found is in exon 21, c.3182T > C:p.Ile1061Thr (I1061T). This variant is the most common associated with NP-C^41^. The first I1061T variant in CaG was found in the RNA-seq data, in a 62-year-old Caucasian female with no known neurological condition. The second I1061T variant comes from the exome-seq data and was found in a 54-year-old Caucasian, who did not mention any neurodegenerative problems. The two individuals with the I1061T variant were also heterozygous. Next, we wanted to see if both the variants identified as pathogenic in our cohort were more frequent in the Quebec population compared to the European population.

### A higher frequency of pathogenic variants in Quebec compared to Europeans

**Figure 1:**
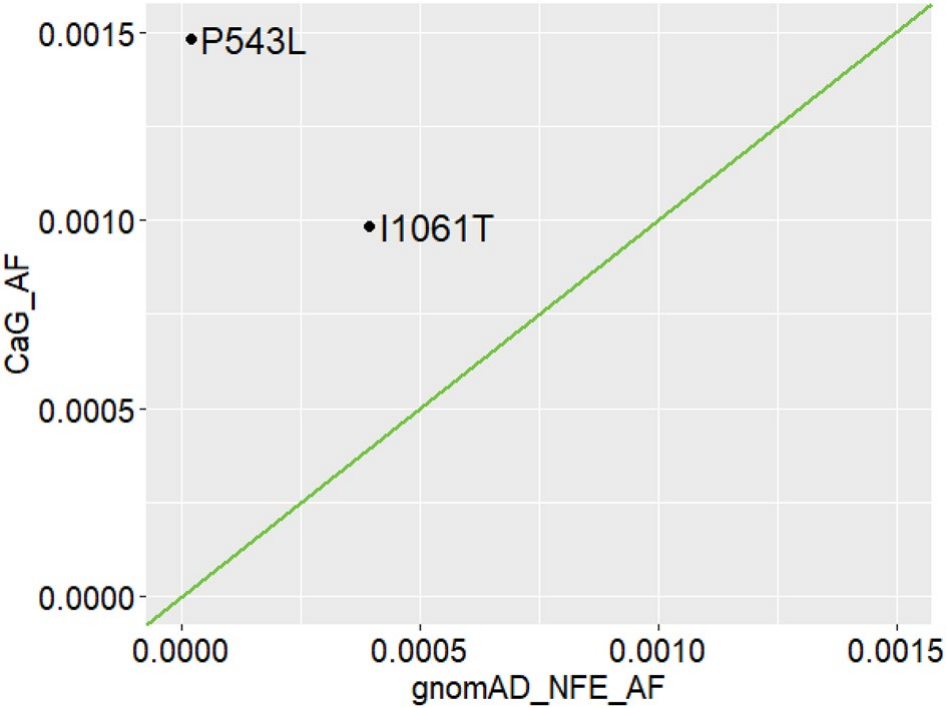
Comparison of variant allele frequencies (AF) in the CARTaGENE cohort compared to gnomAD NFE. The AF of the two identified pathogenic variants, P543L and I1061T is plotted in the CaG cohort (CaG_AF) as a function of the allele frequency in gnomAD NFE (gnomAD_NFE_AF). Made with the ggplot package in R (version 4.1.0).

In total, we had a cohort of 1,016 individuals or 2032 alleles. The P543L variant found in three heterozygotes individuals has an allele frequency (AF) of 1.48e−03 in the CaG cohort and in Non-Finnish Europeans (NFE) from the gnomAD database, the AF is 1.79e−05. The I1061T is observed in two heterozygotes which means an AF of 9.84e−04 and 3.94e−04 in CaG and gnomAD NFE, respectively. Both variants show enrichment in allele frequency in our cohort compared to gnomAD NFE as shown in Fig. 1. The P543L variant is a lot more common in our population (one-sided Fisher exact test p-value = 5.577e−5; Odds Ratio (OR) = 82.3) and the I1061T variant is also enriched although not significantly (p-value = 0.1985; OR = 2.5).

### Estimated prevalence of NP-C in the Quebec population

Using the allele frequency from both pathogenic variants, P543L and I1061T, we estimated the prevalence of NP-C in the Quebec population. The frequency of allele mutant (q) was 0.0025 (5/2032) and the frequency of the disease (q^2^) is 6.055e−6. Assuming Hardy–Weinberg equilibrium, there should be 0.51 new case of NP-C every year in Quebec based on the 84,200 births in 2019^42^. We estimate a prevalence of 0.61 per 100,000 births in Quebec.

## Discussion

In this study, we defined the prevalence of NP-C in the Quebec population. Analysis of RNA- and exome-sequencing from the CaG cohort led to the identification of two pathogenic variants P543L and I1061T. The allele frequencies of these variants were used to calculate the prevalence as well as establish if the variants were enriched in our population.

### Pathogenic variants

The P543L variant was previously seen in one infantile homozygote French-Canadian with severe neurological symptoms reminiscent of classical infantile NPC phenotype and passed away at age 6^39^. Although the study described genetic and clinical findings in 35 patients of different ethnicities, this variant was only found in the French-Canadian patient, suggesting that the P543L variant could be more prevalent in Quebec. In our cohort, P543L was found in three heterozygotes individuals all originating from the same region in Quebec. Those individuals shared a large haplotype that suggests a common French-Canadian ancestor. Since the genotype of the parents of these individuals is not available, phasing of the haplotype is not possible, and thus a direct link cannot be established. However, the French-Canadian population, and specifically the Quebec-Charlevoix region, is well known for founder effects and several diseases or variants have been described more frequently in this region^16,40^. In addition, the P543L variant was significantly enriched in our cohort when compared to Non-Finnish Europeans. Thus, the suggestive haplotype, the enrichment in our population, and the report of this variant in a French-Canadian patient support P543L as a founder mutation. The association of this variant with a regional founder effect is of great importance as it will inform future genetic testing, where this variant should be prioritized in patients presenting a NP-C phenotype in Quebec.

The second pathogenic variant identified, I1061T, is the most prevalent variant associated with NP-C to date. Its prevalence varies depending on the population and the highest, 20%, is found in Western Europe (mainly in France and the UK), followed by Spain (10%), Portugal (6%) and Italy and Germany (5%)^4,41,43^. This variant was also very prevalent in the Hispanic population of the Upper Rio Grande in the United States. This high prevalence is due to a founder effect from the Spanish settlers in Mexico at the beginning of the eighteenth century^41^. Since Quebec was founded by French immigrants in 1600–1700 and English settlers later on, it would explain why this variant is also prevalent in our cohort. On the other hand, the prevalence of I1061T does not significantly differ from that of Non-Finnish Europeans.

### Prevalence of NP-C

In the world, we observe an heterogenous estimation of NP-C prevalence, depending on the population. In Canada, and more specifically in Quebec, no prevalence had been calculated or estimated for NP-C. Elsewhere in the world, the prevalence of NP-C for 100,000 live births is 0.47 in Australia^44^, 0.91 in Czech Republic^15^, 2.2 in Northern Portugal^4^, 0.35 in the Netherlands^3^, 0.82 for France^5^, 0.78 for the United Kingdom^45^ and 1.12 for the United States of America (USA)^46^. By combining these different prevalences, we obtain an average of 0.95 cases per 100,000 births. In all studies except for the USA, prevalence was measured using the number of patients diagnosed with NP-C divided by the number of births in the same period, reported on 100,000 births. For the USA, the prevalence is derived from an estimate based on four databases. The low prevalence observed in Australia and the Netherlands (0.47 and 0.35 per 100,000 respectively) could be explained by an underdiagnosis in the 1990s, when the phenotype spectrum was not clearly defined. More recent prevalence estimates were not found for those countries. Several factors, such as the inclusion of prenatal cases or of heterozygotes can influence prevalence. For example, in France, adding prenatal cases increases prevalence from 0.82 per 100,000 births to 0.96 per 100,000 births^5^. In the UK, many heterozygote individuals for the I1061T variant had neurological symptoms in all age groups^45^. NP-C in the heterozygous form may predispose patients to a late-onset form of the disease with symptoms of dementia, tremors similar to Parkinson’s disease or psychosis^13^. Individuals who are heterozygous for pathogenic variants and who present with atypical symptoms are likely to be misdiagnosed and this could underestimate the true prevalence of NP-C.

As there is no registry of NP-C cases in Quebec and it is impossible to measure the prevalence using the classical method, we used the Hardy–Weinberg equation. We also calculated the prevalence if one NP-C case was born in Quebec every year, every two years, every three years, or every four years. Using this method, the prevalence at birth would be, respectively, 1.19:100,000, 0.59:100,000, 0.39:100,000 or 0.30:100,000. Our prevalence estimate based on the two pathogenic variants found in the CaG cohort is 0.51 case every year or 0.61 case per 100,000 births. Based on clinical data, less than one case appears to be diagnosed in Quebec each year. If one NP-C case was diagnosed every two years, the disease would probably not be underdiagnosed in the province. Otherwise, it would suggest that NP-C could be underdiagnosed clinically in the Quebec population. Since our estimation is only based on heterozygote asymptomatic carriers of two pathogenic variants, it could explain the low estimated prevalence in Quebec. The death of early infantile cases may also play a role in an underestimation. Considering the founder effect, it would be interesting to measure the prevalence of NP-C using data only from individuals from French-Canadian origin in the CaG cohort. It could help us reinforce the role of the P543L variant as a founder mutation in French-Canadians. In the future, establishing a registry of NP-C cases in Quebec is of great importance since it would allow a more precise estimation of the prevalence. It is also important to ensure that all NP-C cases are properly diagnosed in order to start treatment as soon as possible. It is particularly true for adult-onset cases who are more difficult to diagnose but for whom therapy has proven helpful to reduce neurological symptoms^47^. Identifying atypical cases is also a priority to ensure a better understanding of the causes and mechanisms underlying this rare disease.

Genetic testing is readily available for NP-C, for both *NPC1* and *NPC2* genes. In clinical practice, it is generally used as a confirmatory test after initial screening by measuring levels of oxysterols^48^. The level of oxysterols is only done when clinical features suggest the diagnosis of NP-C. Some clinicians use a Suspicion Index tool based on visceral, neurologic and psychiatric symptoms to evaluate the suspicion of NP-C^49^. In Quebec, newborn screening includes cystic fibrosis, congenital hypothyroidism, hemoglobinopathies and several metabolic disorders^50^. The disease prevalence of NP-C remains relatively low but is more prevalent than some disorders that are systematically screened. Given the founder effect in Quebec, the difficult diagnosis and the potential treatment strategies that can improve outcomes, it would be interesting to consider adding oxysterols to blood screening in newborns. One important pitfall to screening with oxysterols is the risk of false positive in patients with neonatal cholestasis^51^. Thus, the optimal screening method for NP-C in this population requires further investigation. There is currently a pilot study of newborn screening assays in New York, including bile acids for NP-C, which will inform us on the clinical validity of screening newborns for complex disorders^52^.

## Conclusion

Certain diseases can be misdiagnosed for a variety of reasons including lack of awareness and heterogeneous clinical presentation. Being able to measure disease prevalence is very important to increase awareness among clinicians. Unfortunately, this can be difficult for rare diseases, where one misdiagnosis can have an important effect on prevalence. For hereditary diseases, estimating prevalence genetically can help compensate for these difficulties and NP-C is an excellent example of a condition for which symptom heterogeneity can make the diagnosis challenging. By identifying a founder variant in the Quebec City region and obtaining data suggesting that the disease is probably underdiagnosed, we are able to encourage clinicians of that area to consider the NP-C diagnosis more readily. This will hopefully lead to earlier detection and treatment of the disease.

## Data Availability

Data was obtained from the CARTaGENE database.

## Code availability

All software used are listed in patients and methods. Custom scripts are available on demand.
